# Role of Alloying Additions in Glass Formation and Properties of Bulk Metallic Glasses

**DOI:** 10.3390/ma3125320

**Published:** 2010-12-21

**Authors:** Na Chen, Laura Martin, Dmitri V. Luzguine-Luzgin, Akihisa Inoue

**Affiliations:** 1 WPI Advanced Institute for Materials Research, Tohoku University, Sendai 980-8577, Japan; E-Mails: dml@wpi-aimr.tohoku.ac.jp (D.V.L.); ainoue@imr.tohoku.ac.jp (A.I.); 2 Department of Materials Science and Metallurgy, University of Cambridge, Pembroke Street, CB2 3QZ, UK; E-Mail: lm439@cam.ac.uk; 3 Institute for Materials Research, Tohoku University, Sendai 980-8577, Japan

**Keywords:** alloying additions, bulk metallic glass, glass formation

## Abstract

Alloying addition, as a means of improving mechanical properties and saving on costs of materials, has been applied to a broad range of uses and products in the metallurgical fields. In the field of bulk metallic glasses (BMGs), alloying additions have also proven to play effective and important roles in promoting glass formation, enhancing thermal stability and improving plasticity of the materials. Here, we review the work on the role of alloying additions in glass formation and performance improvement of BMGs, with focus on our recent results of alloying additions in Pd-based BMGs.

## 1. Introduction

Metallic glasses are metallic solids, but different from conventional crystalline alloys in both structure and properties. Structurally they are characterized by not having a long-range atomic order and therefore, they behave like liquids when they enter the supercooled liquid region (SLR). Yet their structure when solidified is not completely disordered, but contains short- to medium-range ordered clusters. This structure is distinct from crystalline materials and endows metallic glasses with remarkable mechanical, physical as well as chemical, properties. More rigid at lower temperatures, they can serve as structural materials; plastic at higher temperatures can be shaped into very complex forms even of nano-scale size.

In the 1950s, electrodeposited Ni-P and Co-P amorphous films were successfully synthesized by Brenner *et al*. [1,2]. After that, Buckel and Hilsch reported the formation of vacuum deposited amorphous films [3]. In 1960, Duwez *et al*. at Caltech, U.S. reported the formation of Au_75_Si_25_ metallic glass by rapid quenching of a liquid [4]. This pointed the way for a new and simplified method for preparation of alloys in the glassy state, with many metallic glasses being produced as thin sheets, droplets or ribbons. The limited geometries prohibited further study on their mechanical properties and restrained their wide spread use. There are metallic glasses that can be pushed beyond those few geometries and can be produced at a size greater than 1 mm in all three dimensions, they are termed bulk metallic glasses (BMGs). For these BMGs, expectations are high that they will be used as structural and functional materials [5,6,7,8].

The first BMG was reported in 1974 by Chen, who created a Pd-Cu-Si glassy rod with a diameter of up to 1 mm [9]. Then, in the early 1980s, the Turnbull group successfully developed the famous Pd-Ni-P glass forming alloy [10]. This alloy can be prepared at the centimeter-scale by using a boron oxide fluxing method to suppress heterogeneous nucleation [11]. In the late 1980s, Inoue group developed many new glass forming alloys based on common metallic elements including La-, Mg-, Zr-based BMGs [12,13,14]. Simultaneously, Johnson group developed a good glass former Zr-Ti-Cu-Ni-Be alloy [15]. These innovative developments for BMGs have spurred extensive exploration and studies in this field.

In addition to these multi-component glass forming alloys, several binary BMGs including Ni-Nb [16,17], Ca-Al [18], Cu-Zr [19,20,21,22], Pd-Si [23], Cu-Hf [24], Ti-Co [25] and Ni-Ta [26] alloys have been successfully synthesized. This means that even very simple alloys, if subjected to suitable production techniques, can also be prepared in forms of BMGs. But these binary alloys exhibited reduced glass forming ability (GFA) compared to those consisting of more constituents, which was evidenced by their relatively high critical cooling rates and smaller critical sizes for glass formation. Based on these binary BMGs, new BMGs with much larger GFA can be designed through suitable alloying additions. This has instigated the approach of alloying additions to enhance the GFA of the glass forming alloys.

Alloying additions have always been an important metallurgical technique for developing new metallic crystalline materials [27,28]. Materials can be tailored in structure and properties through proper alloying additions, which have been widely accepted as a means of improving mechanical properties and have been applied to a broad range of uses and products in the steel industry. For example, high strength structural steels can be produced due to ultrafine grained microstructure induced by minor alloying additions of Ti, V or Nb. With the addition of 0.1 wt. % B, the room temperature ductility of Ni_3_Al was dramatically increased to 53.8% [29], marking the invention of ductile intermetallic alloys.

The use of alloying additions has been extended to the field of BMGs. Many of the present BMGs can be regarded as developments of binary or ternary alloys as listed in Table 1. The GFA is representative by critical cooling rate *R_c_* for glass formation, which is a direct criterion for evaluating the GFA. The smaller *R_c_* is, the larger GFA. As shown in Table 1, *R_c_* shows a clear decrease with alloying additions, indicating a significant improvement in the GFA. Therefore, proper alloying additions are very effective in promoting glass formation in metallic alloys.

**Table 1:** The critical cooling rate *R_c_* for the selected bulk metallic glasses (BMGs).

Based type	Alloy	*R_c_* (K/s) [Ref.]
Zr-	Zr_66_Al_8_Ni_26_	67 [30]
	Zr_66_Al_8_Cu_12_Ni_14_	23 [30]
La-	La_55_Al_25_Ni_20_	68 [31]
	La_55_Al_25_Ni_5_Cu_15_	36 [31]
	La_55_Al_25_Ni_5_Cu_10_Co_5_	19 [31]
Cu-Zr-	Cu_50_Zr_50_	250 [32]
	Cu_46_Zr_46_Al_8_	40 [32]
	Cu_46_Zr_46_Al_7_Gd_1_	10 [32]
Pd-	Pd_81_Si_19_	100 [33]
	Pd_77.5_Cu_6_Si_16.5_	40 [34]
Ni-	Ni_81_P_19_	>10^6^ [35]
	Ni_40_Pd_40_P_20_	128 [36]
	Ni_10_Cu_30_Pd_40_P_20_	1.57 [36]

The addition of yttrium can significantly improve the GFA of Fe-based BMGs by adjusting the compositions closer to the eutectic point and scavenging any oxygen impurity [37]. With an addition of as little as 1.5% yttrium, a fully amorphous structure can be obtained in (Fe_44.3_Cr_5_Co_5_Mo_12.8_Mn_11.2_C_15.8_B_5.9_)_98.5_Y_1.5_ alloy with a diameter up to 12 mm [38]. The yttrium addition not only destabilizes the competing crystalline phases, but also stabilizes the liquid phase of the (Fe_44.3_Cr_5_Co_5_Mo_12.8_Mn_11.2_C_15.8_B_5.9_)_98.5_Y_1.5_ alloy; this favors glass formation both kinetically and thermodynamically [38]. Minor yttrium additions also show remarkable effect on the GFA of the Cu-Zr-Al alloys, lowering the alloy liquidus temperature and making the composition closer to a quaternary eutectic [39,40]. A blind excessive yttrium addition, however, can lead to drastic deterioration in the GFA of Cu-Ti-based alloys [41]. Doping 1% boron in Ni-Nb-Sn alloy was found to have significant effect on the GFA [42], while the same 1% alloying addition markedly deteriorated the GFA of the Zr_57_Nb_5_Cu_15.5_Ni_12.5_Al_10_ alloy [43]. These experimental results highlight that alloy additions must be chosen carefully as their effect on glass formation and thermal stability of the alloy are not always beneficial.

Alloying additions are a powerful method for improving, and even controlling, the mechanical properties of BMGs by modulating the microstructure. Co-Fe-based ultrahigh-strength BMGs show a fracture strength of 5,180 MPa, which is about five times those of stainless steel and Ti alloys [44]. However, there is little chance they will have any practical applications as structural materials since the Co-Fe-based BMGs show almost no plasticity. The deformation of conventional BMGs is limited in very narrow shear bands, which results in work softening and catastrophic fracture. Such brittle fracture of most of the BMGs is not desirable and limits their wide-spread use. In order to improve the plasticity of BMGs, many approaches have been tried. Among them, alloying additions have proven to be effective in enhancing the ductility of brittle BMGs [45,46,47,48].

In this paper, we summarize recent studies of the role of alloying additions in glass formation and properties of BMGs. So far a variety of elements have been chosen for alloying additions. The beneficial effects of alloying additions can mainly be summarized in two aspects: (1) Improving the GFA; and (2) enhancing the properties, including improving the thermal stability of the glassy structure, strengthening the BMGs, improving the ductility, improving the magnetic properties and corrosion-resistance, *etc*.

## 2. Improving Glass Forming Ability

### 2.1.Alloying with Metalloid Atoms

Small metalloid atoms C, B, and Si have frequently been chosen as alloying additions in BMGs. The effects of metalloid element additions on the thermal stability of the glass forming alloys are summarized in Table 2.

Only 1% C addition in Zr_41_Ti_14_Cu_12.5_Ni_10_Be_22.5_ BMG can further extend the supercooled liquid region (SLR) ∆*T* (defined as *T_x_*-*T_g_*) from 60 K to 90 K [49]. This indicates that the C addition makes the amorphous alloy more thermodynamically stable. Minor B addition also shows remarkable effects on the GFA and thermal stability of Ni-based BMGs [42]. Minor Si addition can increase the maximum thickness of glassy ingots based on Cu-Ti-Zr-Ni from 4 mm to 7 mm [50]. For Ni-based BMG [51], Si also has a great effect on promoting glass formation. 2.5% Si addition can dramatically give rise to a large extension of the SLR of Fe-based metallic glasses, suggesting an improvement in the thermal stability of the glassy phase [52]. These results show that Si is a good choice for alloying in BMGs. Especially in most of the Pd-based BMGs, Si is a main constituent element, demonstrating its important role in the glass formation of these alloys [23,33].

**Figure 1:**
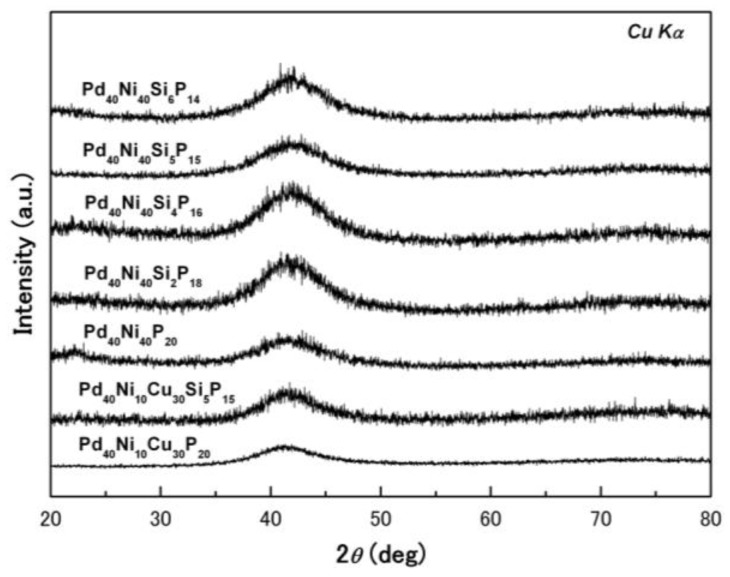
XRD patterns of Pd_40_Ni_40_Si_x_P_20−x_ (x = 0–6) and Pd_40_Ni_10_Cu_30_Si_x_P_20−x_ (x = 0 and 5).

Figure 1 shows the x-ray diffraction (XRD) patterns of the as-prepared Pd_40_Ni_40_Si_x_P_20-x_ (x = 0–6) and Pd_40_Ni_10_Cu_30_Si_x_P_20−x_ (x = 0 and 5) alloys with a diameter of 2 mm. Except for the broad diffraction peaks, no distinctive sharp diffraction peak is observed in the XRD spectra, indicating that all the samples are composed of a glassy phase without any crystallites.

**Figure 2:**
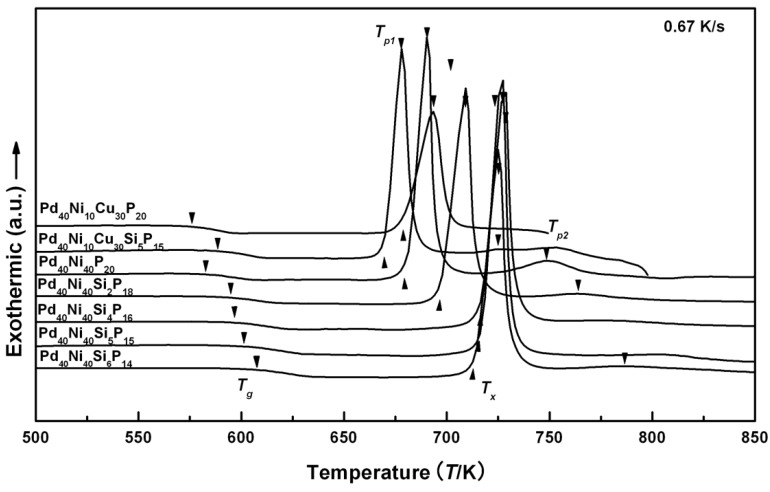
DSC curves of Pd_40_Ni_40_Si_x_P_20−x_ (x = 0–6) and Pd_40_Ni_10_Cu_30_Si_x_P_20−x_ (x = 0 and 5).

Figure 2 shows the differential scanning calorimetry (DSC) traces of Pd_40_Ni_40_Si_x_P_20−x_ (x = 0–6) and Pd_40_Ni_10_Cu_30_Si_x_P_20−x_ (x = 0 and 5) glassy alloys. The thermodynamic and kinetic parameters derived from the DSC curves are listed in Table 3. It can be seen that with Si addition, the glass transition temperature *T_g_* clearly shifts to a higher temperature for both Pd-Ni-P and Pd-Ni-Cu-P alloys. The increased *T_g_* means an increase in viscosity at a given temperature and in the difficulty of inter-atomic diffusion. On the other hand, the initial crystallization temperature *T_x_* firstly increases from 679 K for Pd_40_Ni_40_P_20_ alloy to 716 K for Pd_40_Ni_40_Si_5_P_15_ alloy, and then decreases to 713 K for Pd_40_Ni_40_Si_6_P_14_ alloy as listed in Table 3. Thus, the width of the SLR of Pd_40_Ni_40_Si_x_P_20−x_ glassy alloys firstly increases from 96 K for Pd_40_Ni_40_P_20_ alloy to 120 K for Pd_40_Ni_40_Si_4_P_16_ alloy, and then decreases to 95 K for Pd_40_Ni_40_Si_6_P_14_ alloy.

The width of ∆*T* reflects thermal stability of the supercooled liquid and is also associated with the GFA of some metallic glasses. For most of the BMGs, the thermal stability of liquid phase agrees well with the GFA of the alloys. The more stable the liquid phase, the higher crystallization-resistance the alloy shows. It is easier for a highly stable liquid melt to obtain large undercooling, which favors glass formation upon cooling.

**Figure 3:**
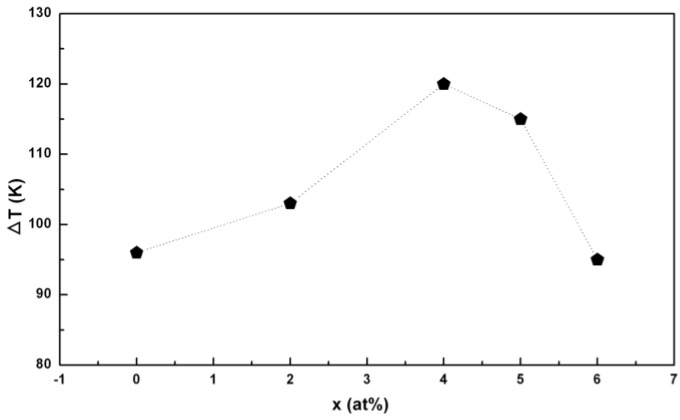
Variation of ∆*T* with increasing Si content.

As shown in Figure 3, the ∆*T* increases with Si additions from 96 K to 120 K and then decreases to 95 K for the Pd_40_Ni_40_Si_x_P_20−x_ (x = 0–6) alloys. It is indicated that only proper Si additions (in this case less than 6%) can improve the thermal stability of the glassy alloys, which reflects an intrinsic feature of the metallic glasses: The high sensitivity of GFA and properties to compositions. It can explain why only a 1% C addition dramatically extended the ∆*T* of the Zr-Ti-Cu-Ni-Be alloy from 60 K to 90 K [49].

The ∆*T* value of Pd_40_Ni_40_Si_4_P_16_ alloy is the largest among these alloys, suggesting that the alloy may have the most stable supercooled liquid. Moreover, the DSC trace of the Pd_40_Ni_40_P_20_ alloy shows a two-stage crystallization behavior. It is clear that the second exothermic peak is much weaker than the first one, showing that the composition Pd_40_Ni_40_P_20_ does not reach, but is close to, the eutectic point. The Si additions make the second peak weaker and weaker and finally lead to its disappearance. Crystallization takes place through a single exothermic reaction in Pd_40_Ni_40_Si_x_P_20−x_ (x = 4 and 5) glassy alloys. It can be concluded that optimum amounts of Si additions (4 at. % or 5 at. %) have adjusted the composition to be closer to a eutectic, thus enhancing the GFA and the thermal stability of Pd_40_Ni_40_P_20_ BMG.

Based on the above experiments, the proper Si addition is ~5% for the glass forming Pd_40_Ni_40_P_20_ alloy. This optimum addition can adjust the composition closer to the eutectic, which stabilizes the supercooled liquid. The same amount of Si addition, however, deteriorates the GFA of the Pd_40_Ni_10_Cu_30_P_20_ alloy. The width of the SLR for the Pd-Ni-Cu-P alloy shows a reduction of 21 K with 5% Si addition. As shown in Figure 2, the quaternary Pd_40_Ni_10_Cu_30_P_20_ alloy shows only one exothermic reaction, suggesting this composition is very close to the eutectic. With Si addition, an extra exothermic peak appears, clearly showing the deviation of the original composition away from the eutectic. So it is suggested that the specific amount of additions depends strongly on the base alloy compositions and has beneficial effects provided that the compositions can be adjusted to closer to the eutectic.

**Table 2:** Summary of the role of optimum amounts of metalloid element additions in various BMGs.

Alloying element	Optimum content x (at. %)	Base alloy (at. %) Ref.	∆*T* (K)		
			without	with	extension
C	1	Zr_41_Ti_14_Cu_12.5_Ni_10−x_Be_22.5_C_x_ [49]	60	90	30
	5	(La_55_Al_25_Ni_20_)_100−x_C_x_ [53]	58 [54]	78	20
	10	Fe_42_Ni_28_Zr_10_C_x_B_20−x_ [55]	-	-	Enhanced
B	1	Ni_60_Nb_36_Sn_4−x_B_x_ [42]	42	58	16
	<3	(Zr_57_Ti_5_Cu_20_Ni_8_Al_10_)_100−x_B_x_ [43]	-	-	Negative effect
	1.5	(La_55_Al_25_Ni_20_)_100−x_B_x_ [53]	58 [54]	-	Enhanced
	1.6	(Fe_81.1_C_13.8_Si_5.1_)_100−x_B_x_ [56]	-	-	Enhanced
	27.5	Fe_91-x_Zr_5_B_x_Nb_4_ [57]	0	43	Enhanced
	0.05	Cu_50_Pr_30_Ni_10_Al_9.9_Ti_0.05_B_0.05_ [58]	-	-	Enhanced
Si	1	(Zr_57_Ti_5_Cu_20_Ni_8_Al_10_)_100−x_Si_x_ [43]			Negative effect
	1	Cu_47_Ti_34−x_Zr_11_Ni_8_Si_x_ [50]	33	58	25
	3.5	Ni_42_Ti_20_Zr_25−x_Al_8_Cu_5_Si_x_ [51]	55	72	18
	2.5	Fe_77_Ga_3_P_12−x_C_4_B_4_Si_x_ [52]	28	48	20
	5	Ni_57_Zr_20_Ti_23−x_Si_x_ [59]	0	60	60
	1	Cu_55-x_Hf_25_Ti_20_Si_x_ [60]	30	60	30
	1	(Cu_0.5_Zr_0.425_Ti_0.075_)_99−x_Si_x_ [61]	40	48	8
	2	Ti_40_Zr_10_Cu_40-x_Pd_10_Si_x_ [62]	50	65	15
	<0.5	(Cu_45_Zr_45_Ag_10_)_100−x_Si_x_ [63]	73 [64]	-	Enhanced
	1.5	(Zr_47_Cu_44_Al_9_)_100−*x*_Si_x_ [65]	60.6	69.6	9
P	4.35	(Fe_81.5_Si_3.8_C_14_Tm_0.7_)_95.65−x_P_x_B_4.35_ [66]	0	64	64

“-“ indicates that no data have been provided by the references.

**Table 3:** Thermodynamic and kinetic parameters and plasticity of Pd_40_Ni_40_Si_x_P_20−x_ (x = 0–6) and Pd_40_Ni_10_Cu_30_Si_x_P_20−x_ (x = 0 and 5) alloys.

	*T_g_*(K)	*T_x_* (K)	*∆T* (K)	*T_p1_* (K)	*T_p2_* (K)
Pd_40_Ni_10_Cu_30_P_20_	576	679	103	693	
Pd_40_Ni_10_Cu_30_Si_5_P_15_	588	670	82	678	726
Pd_40_Ni_40_P_20_	583	679	96	690	749
Pd_40_Ni_40_Si_2_P_18_	594	697	103	709	764
Pd_40_Ni_40_Si_4_P_16_	596	716	120	727	
Pd_40_Ni_40_Si_5_P_15_	601	716	115	726	
Pd_40_Ni_40_Si_6_P_14_	608	713	95	725	787

### 2.2. Alloying with Metallic Atoms

Cu, Au, Ag and Fe atoms have been chosen as alloying additions in Pd-based BMGs [33,36,67,68]. Pd-P metallic glass can only be prepared as ribbons due to the high cooling rate required for glass formation. With Ni addition, the good glass forming Pd_40_Ni_40_P_20_ alloy was developed from the eutectic composition Pd_82_P_18_ alloy [10,11]. By partial substitution of Ni with Cu, the new glass forming alloy Pd_40_Ni_10_Cu_30_P_20_ has been obtained and recognized as one of the best glass formers [36]. A mixture of Au and Ag additions is effective in promoting glass formation in the Pd-Si alloy with a clear extension of the SLR [33]. 2 at. % Au addition to the Pd-Cu-Si-P alloy can extend the SLR to about 80 K [67].

**Figure 4:**
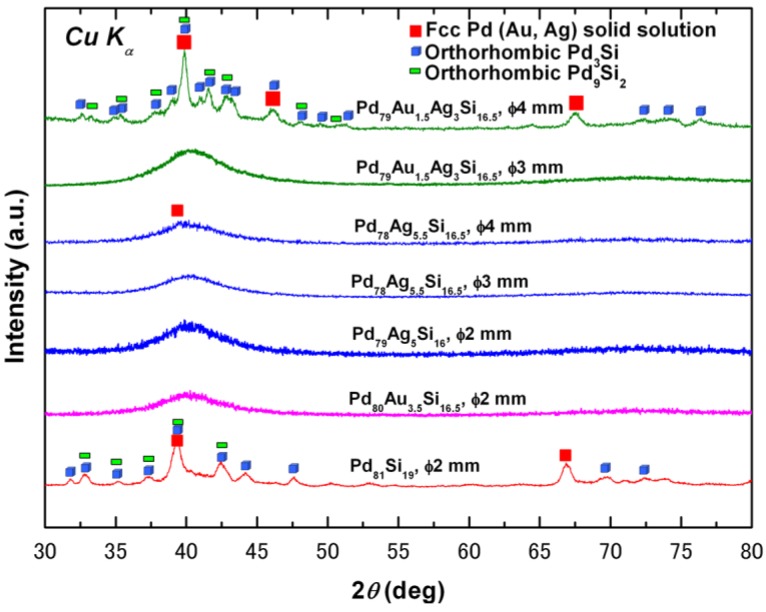
XRD patterns of the Pd-Si-based metallic alloys.

Additions of Au or Ag greatly improved the GFA of the Pd-Si-based alloys and enabled the successful production of bulk metallic glass using copper mold casting as shown in Figure 4. Except for the binary Pd_81_Si_19_ alloy, only broad diffraction peaks characteristic of glass formation were observed in the XRD patterns of the other ternary and quaternary Pd-Si-based alloys with diameters of at least 2 mm. For the Pd_81_Si_19_ alloy, clear sharp diffraction peaks appear, corresponding to the precipitation of nano crystallites identified to be fcc Pd-riched solid solution and the orthorhombic Pd_3_Si and Pd_9_Si_2_ phases. The best glass forming alloy is found to be Pd_79_Ag_5.5_Si_16.5_ alloy, which can form an almost full glass rod with a diameter of 4 mm.

As presented in Figure 4, an fcc Pd rich solid solution is the most stable phase thermodynamically in this system and competes with the glassy phase. Ohkubo and Hirotsu proposed a structural model with fcc Pd-type cluster regions embedded in a dense-random-packing structure of Pd and Si, in an amorphous Pd_82_Si_18_ alloy [69]. It is indicated that the short/medium range ordered units in this alloy have the same symmetry as the translational long-range ordering crystalline phases, which energetically favors crystallization and destabilizes the liquid. Au and Ag can completely form fcc solid solution with Pd, rather than having to introduce more competing intermetallic compounds. Consequently the small amount additions of Au or Ag do not have significant effect in suppressing the precipitation of the fcc crystalline phase. Furthermore, the negative heats of mixing $\Delta H_{Au-Si}^{mix}$ and $\Delta H_{Ag-Si}^{mix}$ are −13 and −6 kJ·mol^−1^, respectively, less than −38 kJ·mol^−1^ of $\Delta H_{Pd-Si}^{mix}$ [70]. Thus, it is easier for Pd to bond with Si and form corresponding metallic compounds, which is in excellent agreement with the crystalline precipitations of Pd_3_Si and Pd_9_Si_2_ phases in the crystallized Pd-Si-based alloys, as evidenced in Figure 4.

The heat for mixing $\Delta H_{Pd-Ag}^{mix}$ is −7 kJ·mol^−1^ while $\Delta H_{Pd-Au}^{mix}$ is 0 kJ·mol^−1^. A negative heat of mixing promotes the local chemical interaction and thus improves the dense atomic packing, which also favors glass formation. This can explain why the best glass forming alloy is Pd_79_Ag_5.5_Si_16.5_ rather than Pd_79_Au_4_Si_17_ alloy. In this study, it is apparent that additions of Au or Ag have improved the GFA of the binary Pd_81_Si_19_ alloys at least when subjected to the conventional copper mold casting. Ruling out the formation of new local configurations, the enhancement of GFA should mainly stem from topological considerations. Au and Ag have the same atomic radii of 0.144 nm, showing an atomic size difference of 23.1% with Si (0.117 nm), larger than the atomic mismatch of 17.1% between Pd (0.137 nm) and Si. Large atomic size difference favors a dense random packing structure and increases the difficulty of atomic rearrangement [6]. Furthermore, the local compressive atomic strains would be generated and destabilize the crystalline structure when larger Au or Ag atoms attempt to occupy the lattice sites through substituting Pd atoms in the crystalline phase. Therefore, the GFA of the Pd-Si alloy has been enhanced both thermodynamically and kinetically by alloying with Au or Ag.

## 3. Enhancing Properties

### 3.1. Enhancing the Mechanical Properties

**Figure 5:**
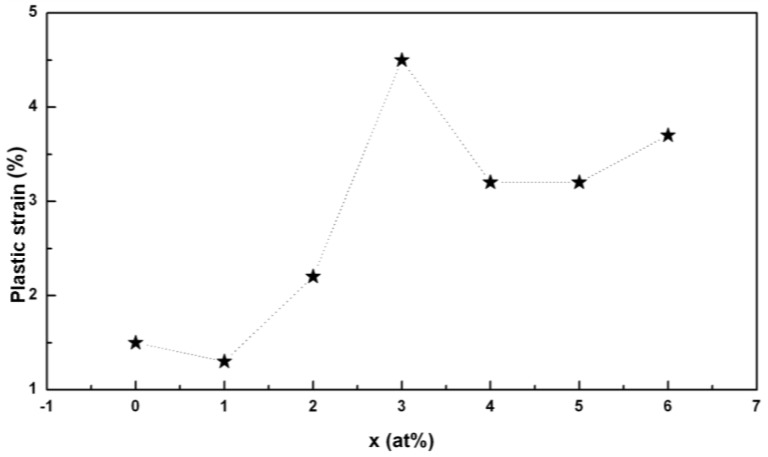
Variation of plastic strain of the Pd_40_Ni_40_Si_x_P_20−x_ (x = 0–6) with Si additions.

**Figure 6:**
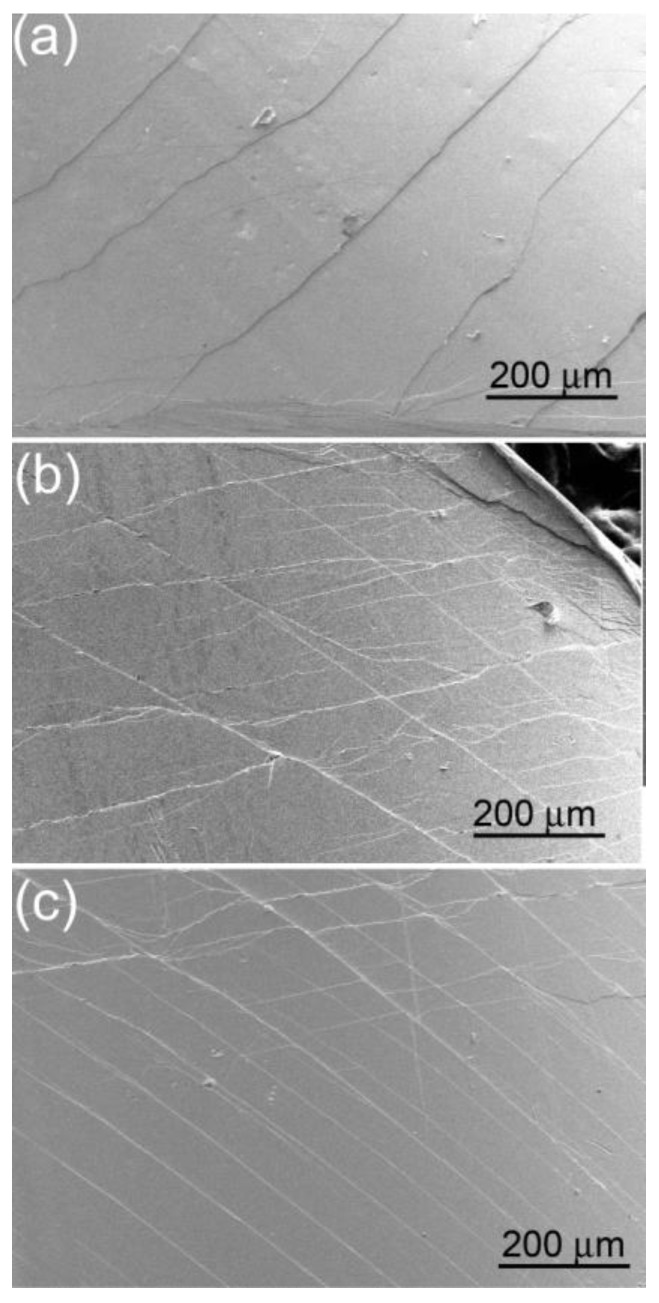
**(a)** SEM image of the deformed Pd_40_Ni_40_P_20_ with a plastic strain of 1.5%; **(b)** SEM image of the deformed Pd_40_Ni_40_Si_4_P_16_ with a plastic strain of 3.2%; **(c)** SEM image of the deformed Pd_40_Ni_40_Si_3_P_17_ with a plastic strain of 4.5%.

Figure 5 shows the variation of plasticity of the Pd_40_Ni_40_Si_x_P_20−x_ (x = 0–6) glassy alloys with Si additions. It is seen that 1 at. % Si addition slightly decreases the plasticity whereas further Si additions result in an improvement in the plastic strain. The maximum plastic strain is 4.5% for the Pd_40_Ni_40_Si_3_P_17_ alloy, three times that for Pd_40_Ni_40_P_20_ alloy (1.5%). Metallic glasses deform inhomogeneously due to highly localized serrated shear deformation. The side views of the deformed BMGs are given in Figure 6, showing the shear band formation corresponding to different strain values for the samples. The ductility of BMGs is closely related to the number of shear bands generated during the deformation process. In general, the more shear bands the samples generate, the larger plasticity the alloys exhibit [71]. It can be seen in Figure 6 that the number of shear bands indeed increases with increasing strain.

Both Ta and Nb are good choices for alloying additions, showing very promising effects on the strength and plastic strain of some BMGs. When 8% Ta was added in Zr-Ni-Cu-Al BMGs, the fabricated BMG matrix composites not only showed high strength (~2.1 GPa), but also displayed dramatically enhanced plastic strain of about 8% prior to failure [72]. The precipitated Ta dendrites distributed very homogeneously effectively as ductile phases, exhibiting good reinforcement in both strength and ductility. Ta additions are also used in Ti-, Ni- and Cu-based BMGs and show prominent roles in glass formation and mechanical properties [73,74,75]. Nb additions can promote the fabrication of Zr_41.25_Ti_13.75_Cu_12.5_Ni_10_Be_22.5_ BMG composites [48]. The Nb dendrites again acted to toughen the BMG composite, which displayed considerable plasticity in both tensile and compressive tests.

Based on the binary Cu-Zr alloy, the new ternary Cu_47.5_Zr_47.5_Al_5_ with 5% Al addition, exhibited a large fracture strain of 18%, simultaneously showing “work-hardenable” behavior in the true strain‑true stress curve [47]. This “work-hardenable” phenomenon contradicted the conventional understanding of the deformation mechanism of BMGs. This work has stimulated increasing interest in the deformation behaviors of ductile BMGs. In the case of Cu-Zr-Al BMG, the “work-hardenable” behavior and large ductility are attributed to an Al addition induced unique structure, correlated with atomic-scale inhomogeneities, leading to an inherent capability of extensive shear band formation and multiplication of shear bands [47]. This indicates that the correct alloying addition can modulate the microstructure at an atomic scale and thus tailor the properties of BMGs.

### 3.2. Controlling the Microstructure and Thus Tuning the Properties

**Figure 7:**
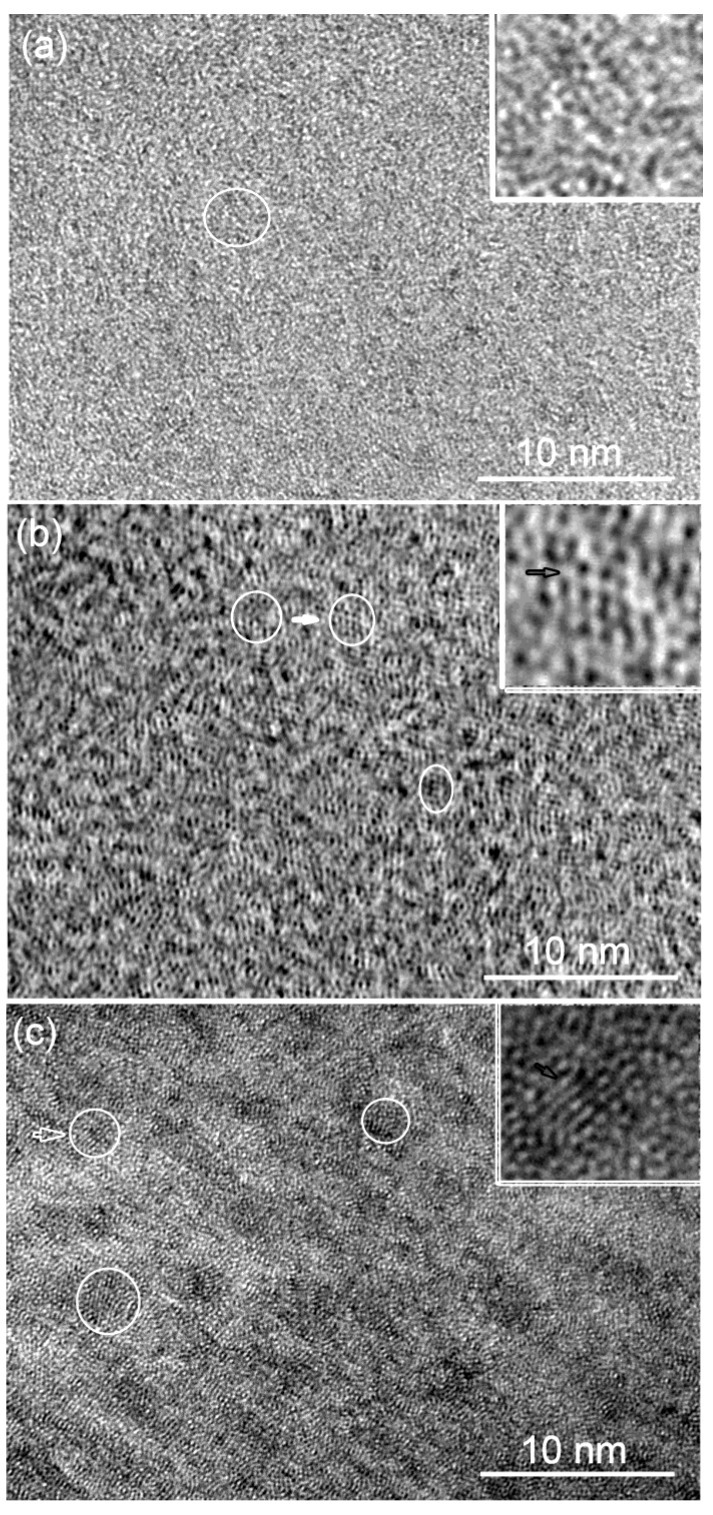
**(a)** HRTEM image of Pd_40_Ni_40_Si_2_P_18_ BMG with a diameter of 2 mm; **(b)** HRTEM image of Pd_40_Ni_40_Si_4_P_16_ BMG with a diameter of 2 mm; **(c)** HRTEM image of Pd_40_Ni_40_Si_6_P_14_ BMG with a diameter of 2 mm.

Minor Si addition is suggested to promote the formation and growth of local clusters in Pd_40_Ni_40_P_20_ BMG, increasing the inhomogeneity in glassy structure, as shown in Figure 7. When 2 at. % Si is added, the structure is typically maze-like glass (see Figure 7a). With increasing Si content, some ordered clusters appear with sizes less than 2 nm as indicated in the inset of Figure 7b. When the Si content reaches 6 at. %, more ordered clusters are embedded in the amorphous matrix as shown in Figure 7c. Furthermore, the contrast of the regions, including these ordering clusters, becomes deeper than the rest of the structure, indicating a nano-scale structural agglomeration in Pd_40_Ni_40_Si_6_P_14_ BMG. The size of these dark regions is about ~5 nm.

In Pd-Ni-Si-P BMGs, local nano-clusters with sizes of 1–2 nm were dispersed in the dense-randomly-packed glassy matrix, as can be seen in Figure 7. If several zones become close enough to be regarded as one union under shear force, they would be of an equivalent size to the shear bands, leading to delocalization, deviation and blunting of the shear bands. Such ordering clusters can also be found in a ductile Ni-based BMG [76]. On the other hand, as presumed in Hf_55_Co_25_Al_20_ glassy alloy, these clusters do not form clear crystallographic arrays but just the ordered regions [77], which trigger the growth of the primary cubic phase. Thus, during the deformation process, these ordering clusters may also function as nucleation sites, and can grow into larger nanoscale crystalline phases induced by local heating, leading to nano crystals toughening of the BMGs [78].

In other BMGs, alloying additions also play a very significant role in their deformation behaviors [67]. Pd_79_Cu_4_Au_2_Si_10_P_5_ BMG exhibited a plastic strain over 13%, much larger than conventional BMGs [67]. In addition to the effect on mechanical properties of BMGs, alloying additions also show substantial effects in improving the magnetic properties and enhancing the corrosion resistance of the glass forming alloy systems.

Minor Ni and Cr additions can be used to improve the soft magnetic property of Fe-based BMGs [79,80], which holds promise in applications as magnetic conductors or magnetic elements. With a small amount of Cr, Mo or W additions, Cu-based BMGs exhibited a superior corrosion resistance [80]. Alloying of Nb and Hf has also proven to show beneficial effects on the corrosion resistance and thermal stability of Zr-Cu-based BMGs [81,82]. The enhancement of corrosion resistance is ascribed to the formation of highly protective films.

## 4. Understanding the Role of Alloying Additions in Glass Formation

The role of alloying additions in glass formation is usually evaluated by means of both thermodynamic and kinetic considerations. The widely used thermodynamic parameters are the supercooled liquid region ∆*T* and the liquidus temperature *T_l_*. Both reflect the thermal stability of the liquid phase. A large ∆*T* indicates high crystallization-resistance and is attributed to a high GFA for most of the BMGs. *T_l_* is closely related to the kinetic process of nucleation and crystallization. A decrease in *T_l_* enables the liquid phase to obtain larger undercooling, which would slow down the nucleation and crystallization. It can explain why the GFA of BMGs is usually improved provided that alloying additions adjust the composition closer to the eutectic. The kinetic parameters including the critical cooling rate *R_c_* and the viscosity *η* of the supercooled liquid are also good indicators for assessing the effect of the alloying additions. The fundamental criterion for glass formation is to avoid crystallization, indicating that the kinetic processes, involving stabilizing the liquid phase and destabilizing the competing crystalline phases, are the key aspects in glass formation.

### 4.1. Stabilizing the Liquid Phase

It is generally found that the addition elements have large negative heats of mixing with the main components of the glass-forming alloys [27,28,32]. This means that the addition elements show a high tendency to form new local atomic pairs due to strong bonding with the major constituents of the alloys. Furthermore, the local chemical configurations of the clusters in the supercooled liquid are usually different from those of the competing crystalline phases for good glass formers [5,6,7,8]. In order to initiate the nucleation and crystallization, the short range or medium range ordered clusters in the supercooled liquids have to be disturbed. At the same time, long range diffusion of the involved atoms is necessary for the formation of new local ordering clusters as the precursors of the crystalline phases. The new local ordered clusters, induced by alloying additions, can give rise to an additional energy barrier for the supercooled liquid to surpass prior to nucleation. Meanwhile long range atomic inter-diffusion becomes more difficult as more varied elements are involved and the complexity of the interactions is increased. Consequently, it is easier for the liquid phase to retain its disordered structure upon cooling.

### 4.2. Destabilizing the Competing Crystalline Phases

Appropriate alloying additions are expected to improve the GFA through destabilizing the competing crystalline phase formation by introducing atomic scale strains. Glass formation is always a competition process between molten liquid and crystalline phases [27,28]. Alloying additions in the glass forming alloys can either cause a disturbance to the local chemical environment, due to the strong affinity with the base constituents, or increase the compact density of the liquid phase due to their atomic size mismatch. Both effects can lead to a higher viscosity of the liquid alloys and lower diffusivity of the involved atoms. Thus the glass formation is strongly enhanced kinetically. If the concentration of the alloying elements is high enough, alternative competing crystalline phases are triggered between the alloying elements and the matrix constitutes. In this case, the alloying additions would play a detrimental effect on the GFA of the BMGs. Provided that the alloying additions do not change the original competing crystalline structure, the added atoms either substitute the matrix element to occupy the crystalline lattice sites or are located in the interstices. Both substitutional atoms and interstitial atoms introduce large strains in the crystal lattice [82], which greatly destabilize the crystal lattice. Thus, a topological instability for crystalline formation is produced and glass formation is favored.

### 4.3. Scavenging the Oxygen Impurity from the Supercooled Liquid

The oxygen impurity level in the glass forming alloys has been found to play a crucial role in the viscosity and the crystallization kinetics of the supercooled liquids [27,28,32,43,83,84,85,86]. Oxygen triggers the formation of metastable crystalline phases and thus dramatically reduces the GFA of the Zr-based alloys [83,84,85]. Proper alloying additions are effective in suppressing the crystalline phase formation and alleviating the detrimental effect of oxygen.

Yttrium has a strong affinity with oxygen so that an optimum yttrium addition has significant effects on improving the GFA and enhancing the manufacturability by scavenging the oxygen impurity and promoting the formation of innocuous yttrium oxides in Zr-, Fe- and Ti-based alloys [37,43,58,86,87,88]. Besides yttrium, the other rare earth elements such as Gd, Ho, Pr, Nd, Tb and Dy are also a good choice for alloying additions due to the enhanced oxygen resistance in manufacturability of the glass forming alloys [89,90,91].

### 4.4. Strategy for Pinpointing Optimum Alloying Additions

Although it is accepted that alloying additions have a significant effect on glass formation, the underlying mechanisms for the enhancement of GFA is still poorly understood owing to complex interactions among the multi components. Only proper alloying additions, with the correct choice of elements and at an optimum concentration, lead to an enhanced GFA and improved properties of glass forming alloys. Based on experimental results, some empirical rules can be summarized and applied for quantitative selection of alloying elements.

When selected elements are used to substitute the main constituent elements, the elements, belonging to the same groups located in a periodic table as at least one of the main constituent elements involved in the alloys, are preferred. Elements belonging to the same group usually have very similar properties owing to the same electronic configurations in their valence shell. Thus the anticipated effect of this substitution is to introduce higher entropy in terms of the “confusion principle”.

The basic principle for selection of alloying elements, including both metallic and metalloid elements, is to adjust the composition closer to the eutectic by lowering the liquidus temperature, enabling the liquid melts to obtain large undercooling. Spurred by this intent, the amount of the selected elements can be quantitatively determined using some preliminary experiments, e.g., DTA method to obtain the melting behavior of the alloys. Then a proper addition can be pinpointed to be the one inducing the maximum depression of the liquidus temperature. For example, based on the eutectic Ni_81_P_19_ binary alloy, substituting 50% Ni for Pd, the same group element as Ni, significantly enhanced the GFA of this binary alloy and enabled the Pd_40.5_Ni_40.5_P_19_ BMGs fabrication, which is very close to the ternary eutectic.

Certainly there are some exceptions, for example, Ni-Si and Au-Al binary alloys, originally showing very deep eutectic points but exhibiting poor GFA. Fully glassy structure cannot even been obtained using rapid solidification of the liquid melts of these binary alloys. Furthermore, based on the eutectic Ni_81_Si_19_ alloy, substituting 50% Ni for Pd cannot help the successful fabrication of BMGs as it does to Ni_81_P_19_ alloy. So it is also important to select the suitable alloys, based on which, proper alloying additions are really useful for further enhancing the GFA of these based alloys.

## 5. Conclusions

To date a variety of elements have been chosen for alloying additions in BMGs. Dependent on the different alloying additions, the beneficial effects can mainly be summarized as: (1) Enhancing the GFA; (2) enhancing the properties including thermal stability, mechanical, physical and chemical properties of the BMGs.

With alloying additions, glass formation is favored both thermodynamically and kinetically. The first key effect is that the alloying elements, particularly small metalloid atoms, can form new local atomic pairs due to the strong affinity with the major constituents of the alloy. The local topological configurations in the supercooled liquid, introduced by alloying additions, are usually incompatible with the transitional long range ordered crystalline phases. This creates an additional barrier for nucleation of new crystals. Furthermore, the high level atomic scale local strains are induced due to the large atomic size mismatch between the original constitutes and the alloying additions, which greatly destabilizes the crystalline phases. Thus, combining the stabilization of the liquid melts and the destabilization of the competing crystalline phases, the glass formation is strongly enhanced due to alloying additions.

Proper alloying additions are also an effective and simple approach to developing new BMGs with novel properties by tuning the microstructures. It has been shown that alloying additions have significant effects on improving the thermal stability, enhancing the strength and plasticity, improving the magnetic properties and enhancing the corrosion resistance of BMGs.

In conclusion, alloying additions are an effective method to improve the GFA and the properties of BMGs. It is of technological and theoretical importance to apply this simple method to design and develop new BMGs with tailored microstructure and controllable properties.
